# iPLA_2_β Overexpression in Smooth Muscle Exacerbates Angiotensin II-Induced Hypertension and Vascular Remodeling

**DOI:** 10.1371/journal.pone.0031850

**Published:** 2012-02-20

**Authors:** Lindsay E. Calderon, Shu Liu, Wen Su, Zhongwen Xie, Zhenheng Guo, Wanda Eberhard, Ming C. Gong

**Affiliations:** 1 Department of Molecular and Biomedical Pharmacology, University of Kentucky, Lexington, Kentucky, United States of America; 2 Department of Physiology, University of Kentucky, Lexington, Kentucky, United States of America; 3 Department of Internal Medicine, University of Kentucky, Lexington, Kentucky, United States of America; Northwestern University, United States of America

## Abstract

**Objectives:**

Calcium independent group VIA phospholipase A_2_ (iPLA_2_β) is up-regulated in vascular smooth muscle cells in some diseases, but whether the up-regulated iPLA_2_β affects vascular morphology and blood pressure is unknown. The current study addresses this question by evaluating the basal- and angiotensin II infusion-induced vascular remodeling and hypertension in smooth muscle specific iPLA_2_β transgenic (iPLA_2_β -Tg) mice.

**Method and Results:**

Blood pressure was monitored by radiotelemetry and vascular remodeling was assessed by morphologic analysis. We found that the angiotensin II-induced increase in diastolic pressure was significantly higher in iPLA_2_β-Tg than iPLA_2_β-Wt mice, whereas, the basal blood pressure was not significantly different. The media thickness and media∶lumen ratio of the mesenteric arteries were significantly increased in angiotensin II-infused iPLA_2_β-Tg mice. Analysis revealed no difference in vascular smooth muscle cell proliferation. In contrast, adenovirus-mediated iPLA_2_β overexpression in cultured vascular smooth muscle cells promoted angiotensin II-induced [^3^H]-leucine incorporation, indicating enhanced hypertrophy. Moreover, angiotensin II infusion-induced c-Jun phosphorylation in vascular smooth muscle cells overexpressing iPLA2β to higher levels, which was abolished by inhibition of 12/15 lipoxygenase. In addition, we found that angiotensin II up-regulated the endogenous iPLA_2_β protein *in-vitro* and *in-vivo*.

**Conclusion:**

The present study reports that iPLA_2_β up-regulation exacerbates angiotensin II-induced vascular smooth muscle cell hypertrophy, vascular remodeling and hypertension via the 12/15 lipoxygenase and c-Jun pathways.

## Introduction

Phospholipase A_2_ is a large family of enzymes that catalyze the hydrolysis of the sn-2 ester bond on phospholipids to produce free fatty acids (*e.g*., arachidonic acid) and lysophospholipids [1]. Calcium independent group VIA phospholipase A_2_ (iPLA_2_β), a member of the phospholipase A_2_ superfamily, is an intracellular protein that is catalytically active in the absence of calcium [1]. iPLA_2_β was initially implicated as a housekeeping enzyme in phospholipid remodeling; however, accumulating evidence indicates that it also plays fundamental roles in cellular signaling, causing cell activation, proliferation, migration or apoptosis [1]. Moreover, iPLA_2_β plays a significant role in a variety of diseases including neurodegenerative disorders [2], [3], cardiac ischemia induced arrhythmia [4], [5], [6] and tumor genesis and metastasis [7], [8]. However, whether iPLA_2_β is involved in hypertension remains unreported.

An estimated 29% of the United States adult (>18 years of age) population is hypertensive, exposing these individuals to an increased risk of mortality and cardiovascular events over their lifespan [9]. The mechanisms underlying primary hypertension remain incompletely understood. Evidence obtained from studies on cultured cells or isolated tissues demonstrates that iPLA_2_β is involved in the regulation of multiple vascular smooth muscle functions and thus may contribute to hypertension and associated vascular remodeling. We [10], [11] and others [12], [13], [14] have demonstrated that iPLA_2_β plays an important role in vascular smooth muscle contraction regulation. Inhibition of iPLA_2_β by the selective suicidal iPLA_2_ inhibitor BEL, anti-sense oligonucleotides or genetic deletion drastically inhibit arachidonic acid release induced by vasopressin [15], thrombin [16], and thapsigargin/A23187 [17]. Moreover, inhibition or genetic deletion of iPLA_2_β significantly reduce thrombin-induced DNA synthesis [16] and smooth muscle migration and proliferation, which was analyzed in isolated mesentery artery tissue in an explant assay [17], suggesting that iPLA_2_β is involved in smooth muscle migration and proliferation. Together, these data imply that vascular smooth muscle iPLA_2_β is pro-hypertensive. On the other hand, iPLA_2_β is also required for angiotensin II (Ang II) induced RGS2 transcription in vascular smooth muscle cells (VSMC) [18] and deleting iPLA_2_β promotes vascular constriction [14]. Since RGS2 is a negative regulator of Ang II-induced hypertension, these imply vascular smooth muscle iPLA_2_β can also be anti-hypertensive. Taken together, the precise role that vascular smooth muscle iPLA2β plays in hypertension and vascular remodeling *in vivo* remains to be determined.

The activity of iPLA_2_β is regulated at multiple levels including allosteric interaction with ATP [19] or calmodulin [20], covalent modification by acylation [21], and potentially by proteolysis and translocation [22]. Interestingly, recent evidence suggests that, in addition to post-translational regulation, the iPLA_2_β protein level is up-regulated under various pathological conditions; the mRNA and protein are up-regulated in Type 1 diabetic Akita mouse pancreatic cells [23], in astrocytes stimulated with pro-inflammatory lipopolysaccharide [24] and in C2C12 myotubes by ischemia [6]. In particular, we have found that iPLA_2_β is up-regulated in VSMC cultured in the presence of high glucose, and is up-regulated in the vasculature from streptozotocin-induced hyperglycemic rats or Type 2 diabetic db/db mice [11]. However, whether the up-regulated iPLA_2_β, among the numerous alterations present under these pathological conditions contributes to hypertension and vascular remodeling is unknown.

We have developed a smooth muscle specific iPLA_2_β transgenic mouse model to determine whether up-regulated iPLA_2_β modulates Ang II infusion-induced hypertension and vascular remodeling. Ang II is the major bioactive peptide of the renin-angiotensin-aldosterone system and its dysregulation is one of the major factors contributing to the pathogenesis of hypertension. Chronic subcutaneous infusion of Ang II induces hypertension and vascular remodeling and has been used extensively as a model to decipher the mechanisms underlying hypertension. Our *in-vivo* and *in-vitro* studies demonstrate that vascular smooth muscle iPLA_2_β up-regulation exacerbates Ang II-induced hypertension and vascular remodeling.

## Methods

### Ethics Statement

All animal work has been conducted according to relevant national and international guidelines. Animal protocols used in the study were approved by the Institutional Animal Care and Use Committee (IACUC) of the University of Kentucky, approved ID 00920M2005.

### Animals

Smooth muscle specific overexpressing iPLA_2_β transgenic mice (iPLA_2_β-Tg) were generated, characterized, and backcrossed to C57/B6 for over 10 generations as described elsewhere (Liu et.al., manuscript submitted). The expression of iPLA_2_β is driven by a rabbit smooth muscle myosin heavy chain promoter. The 12/15 lipoxygenase knockout mice were purchased from The Jackson Laboratory.

### Materials

Ang II was purchased from Sigma (St. Louis, MO). 17-Octadecynoic acid, MK886, Baicalein, and Luteolin were purchased from Cayman (Ann Arbor, MI). Nordihydroguaiaretic acid and Indomethacin were purchased from Biomol (Plymouth Meeting, PA). The primary antibodies to β-actin, phospho-c-Jun, phospho-p38 MAPK, total-p38 MAPK were purchased from Cell Signaling (Danvers, MA). The iPLA2β antibody was made in our lab, and the generation and characterization of the antibody was previously documented [18].

### Primary Cell Culture

Aortic VSMC were isolated from 12–13 wk old rats or mice and used at passages 5 to 10 as previously described [25].

### Western Blot

After the indicated treatments, cells were collected, lysates were prepared, and the proteins were separated by SDS-polyacrylamide gel electrophoresis (SDS-PAGE), transferred to nitrocellulose membranes, which were then western blotted with the following antibodies: iPLA_2_β (1∶2500), β-actin (1∶2500), phospho-c-Jun (1∶1000), phospho-p38 MAPK (1∶2000), total-p38 MAPK (1∶2000), respectively. The proteins were quantified using the ECL Plus Western Blotting Detection System (GE Healthcare).

### Blood Pressure Measurement

Eight pairs of 13-wk old male iPLA_2_β-Tg and iPLA_2_β-Wt (littermate) mice were anesthetized with isoflurane and implanted with telemetry probes (TA11PA-C10, Data Sciences International, St. Paul, MN) in the left carotid artery. After 7 to 10 days of recovery, basal blood pressure, heart rate, and locomotor activity data were collected continuously using the Dataquest A.R.T. system (Data Sciences International, St. Paul, MN) for 72 h. Osmotic mini-pumps (Alzet Model 2002) were then implanted, subcutaneously, to infuse Ang II (500 ng/kg/min, 14 days) or saline. During the infusion period, blood pressure data was collected continuously for 24 h every other day.

### Morphometric Analysis of Vascular Remodeling

At the 14^th^ day of Ang II or saline infusion, the mice were euthanized and perfused under physiological pressure with PBS and 4% paraformaldehyde. Thoracic aortas (2 mm from descending aorta) and secondary branches of the mesenteric arteries were isolated, cleaned, embedded, and cut into 5 µm sections. Sections were stained with HE (Hematoxylin-Eosin, Surgipath), or Elastin (Elastin Stain kit, Richard-Allan Scientific), or collagen (Masson Trichrome Stain, Richard-Allan Scientific). Images were captured and analyzed using an Olympus digital camera with Olympus MicroSuit-B3 Software. Elastin stained slides were used for morphometric analysis. The media thickness was determined by measuring the distance from the internal elastic lamina (IEL) to the external elastic lamina (EEL). For each slide, measurements from 4 points (12, 3, 6 and 9 o'clock positions) were averaged. The media area was determined by measuring the area between the IEL and EEL. The media∶lumen ratio was calculated based on the measured lumen area and media areas.

### Immunohistochemistry

tlsb

OCT embedded 2^nd^ order mesentery artery branches were sectioned and fixed for 30 min in paraformaldehyde. Sections were blocked with normal goat serum and incubated with anti-p-c-Jun (1∶100). Slides were then washed, incubated in fluorescently-labeled (1∶200, Alexa-Fluor 594, Invitrogen) secondary antibodies and counterstained with DAPI (300 nM).

### iPLA_2_β Adenoviral Infection

The doxycycline-inducible iPLA_2_β-expressing adenoviral vectors were generated and purified as previously described [10]. Serum starved cells were infected for 12 h with Ad-iPLA_2_β (500 MOI) and Ad-Tet-on (2000 MOI). Doxycyclin (1 µg/ml) was added to the culture medium for 24 h to induce iPLA_2_β expression. The cells were then harvested for iPLA_2_β protein expression analysis or used for [^3^H]-leucine incorporation assay.

### [^3^H]-Leucine Incorporation

After the indicated treatment, the VSMC were incubated with [^3^H]-leucine (0.25 µCi/well) for 24 h, washed, harvested and [^3^H]-leucine incorporation was quantified by liquid scintillation spectroscopy (Packard, Packard BioScience Company) as previously described [26].

### Statistical Analysis

Each experiment was repeated a minimum of three times. Data were expressed as mean ± S.E. Statistical analysis was performed by using one- and/or two-way analysis of variance with repeated measurement for multiple groups. A post-hoc Bonferroni analysis was performed when appropriate (GraphPad Prism 4). Statistical significance was set at p<0.05.

## Results

### Smooth muscle specific iPLA_2_β overexpression exacerbates Ang II infusion-induced hypertension without affecting basal blood pressure

**Figure 1:**
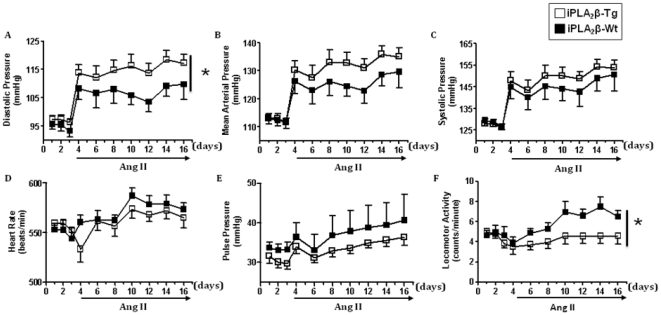
Enhanced diastolic blood pressure increase in iPLA_2_β-Tg mice in response to Ang II infusion. Blood pressure was measured in 13-wk old male, iPLA_2_β-Wt and iPLA_2_β-Tg mice by radiotelemetry. 24 h averages of systolic (**A**), diastolic (**B**), and mean arterial pressure (**C**), heart rate (**D**), pulse pressure (**E**), and locomotor activity (**F**) were collected prior to and during Ang II infusion (500 ng/kg/min, 14 days). n = 8; *: p<0.05 by two-way ANOVA with repeated measures.

To examine the role of iPLA_2_β in basal blood pressure and in Ang II-induced hypertension, we utilized the smooth muscle specific iPLA_2_β-Tg mice that we previously developed (Liu et.al., manuscript submitted). No significant difference was detected in basal systolic (Fig. 1A); diastolic (Fig. 1B) or mean arterial blood pressure (Fig. 1C) between the iPLA_2_β-Tg and iPLA_2_β-Wt mice. Ang II infusion (500 ng/kg/min, 14 days) rapidly increased systolic, diastolic, and mean arterial pressure in both strains (Fig. 1A–C); saline infusion did not affect these parameters (Data not shown). Interestingly, diastolic pressure after Ang II infusion was significantly higher in iPLA_2_β-Tg mice (115±8.6 mmHg) than in the iPLA_2_β-Wt mice (107±10.3 mmHg) (Fig. 1B). Both the systolic and mean arterial pressures were higher in the iPLA_2_β-Tg mice after Ang II infusion, but the difference did not reach statistical significance. Additionally, no difference in the heart rate or pulse pressure was detected and the locomoter activity was lower in the iPLA_2_β-Tg mice than the iPLA_2_β-Wt mice (Fig. 1D–F).

### Vascular remodeling induced by Ang II infusion is exacerbated in iPLA2-Tg mice

**Figure 2:**
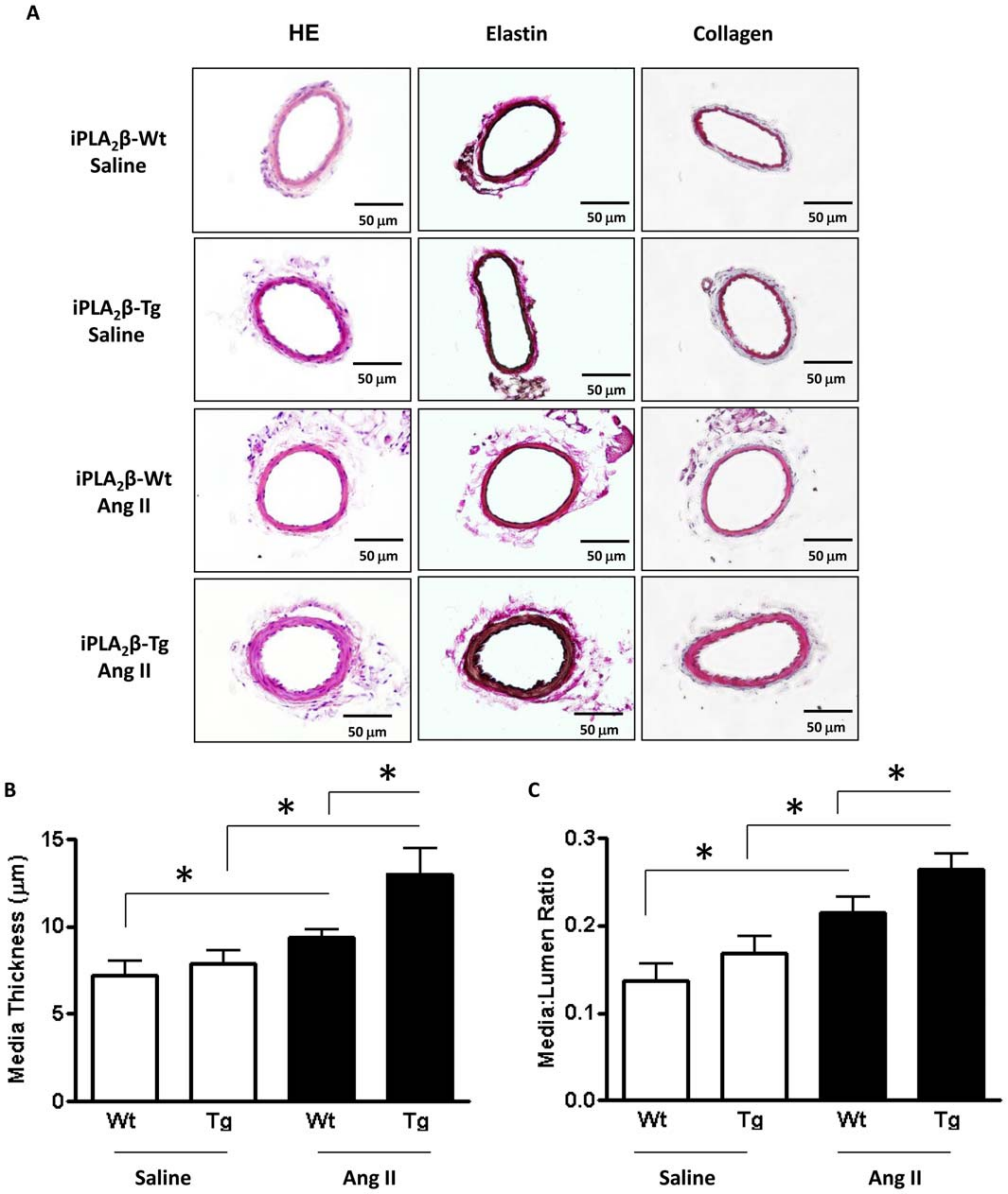
Exacerbated mesenteric artery remodeling in iPLA_2_β-Tg mice in response to Ang II infusion. Vascular remodeling was analyzed in embedded sections of the 2^nd^ order branch of mesenteric arteries from the iPLA_2_β-Wt and iPLA_2_β-Tg mice infused with saline or Ang II (500 ng/kg/min, 14 days). Representative images of vessel sections stained with HE, elastin or collagen (**A**). The media thickness (**B**) and media∶lumen ratio (**C**) were quantified. n = 8, *: p<0.05, **: p<0.01, ***: p<0.001 by one-way ANOVA.

**Figure 3:**
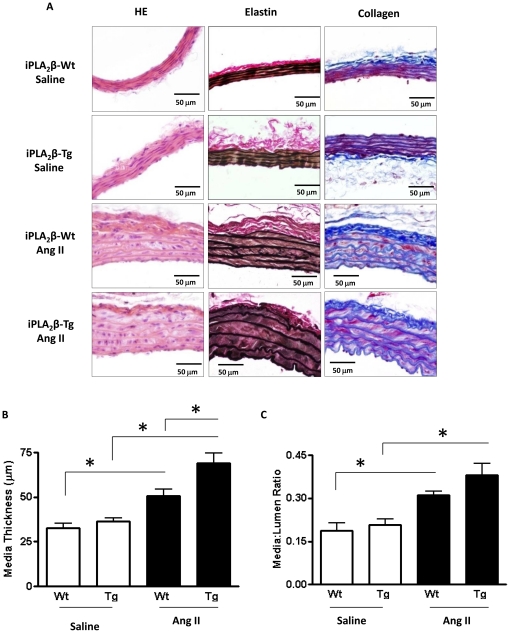
Exacerbated thoracic aorta remodeling in iPLA_2_β-Tg mice in response to Ang II infusion. Vascular remodeling was analyzed in the thoracic aorta of the iPLA_2_β-Wt and iPLA_2_β-Tg infused with Ang II (500 ng/kg/min) or saline. Representative images of vessel sections stained with HE, elastin or collagen (**A**). The media thickness (**B**) and media∶lumen ratio (**C**) were quantified. n = 8, **: p<0.01, ***: p<0.001 by one way ANOVA.

To investigate the mechanism underlying the exacerbation of Ang II infusion-induced blood pressure elevation in iPLA_2_β-Tg mice, we used morphometric analysis to determine whether iPLA_2_β overexpression affects Ang II-induced vascular remodeling. In the mesenteric arteries, iPLA_2_β overexpression alone did not significantly alter mesenteric artery collagen or elastin expression (Fig. 2A). However, iPLA_2_β overexpression significantly promoted the Ang II infusion-induced increase in media thickness (Fig. 2B) and media∶lumen ratio (Fig. 2C). In the thoracic aorta, iPLA_2_β overexpression also significantly promoted Ang II infusion-induced increases in media thickness (Fig. 3B) without altering the media∶lumen ratio (Fig. 3C) and the elastin or collagen expression (Fig. 3A).

### iPLA_2_β overexpression enhances Ang II-induced vascular remodeling by promoting vascular smooth muscle hypertrophy via the 12/15 lipoxygenase pathway

**Figure 4:**
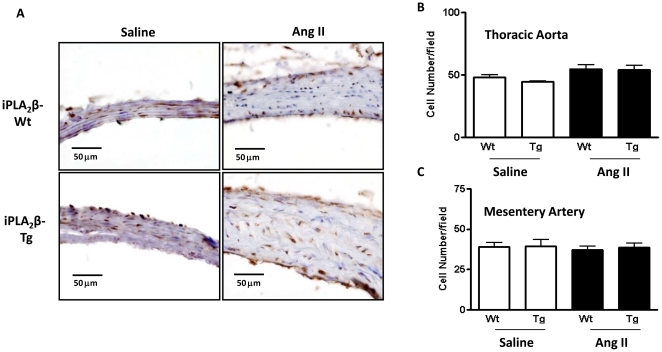
Lack of difference in cell proliferation between iPLA_2_β-Tg and iPLA_2_β-Wt mice by Ang II infusion. Mice were infused with saline or Ang II (500 ng/kg/min, 14 days) and thoracic aortas were isolated and sectioned. (**A**) are representative images of the sections stained with anti-PCNA antibody. (**B**) and (**C**) are summaries of the cell number analysis using HE stained sections. n = 8.

To elucidate the mechanism via which iPLA_2_β enhances Ang II-induced vascular remodeling, we investigated the effects of iPLA_2_β overexpression on VSMC proliferation and hypertrophy. The proliferation state was assessed by PCNA staining and cell counting. No significant difference was detected in PCNA staining [aorta (Fig. 4A) or mesentery artery (data not shown)] or cell number [aorta and mesentery artery (Fig. 4B,C)].

**Figure 5:**
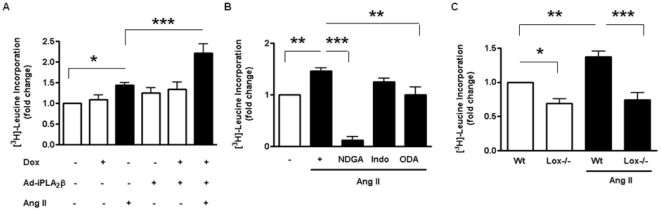
iPLA_2_β overexpression promotes Ang II-induced [^3^H]-leucine incorporation in VSMC via the 12/15 lipoxygenase pathway. [^3^H]-leucine incorporation was quantified by liquid scintillation spectroscopy in cultured iPLA_2_β-Wt VSMCs treated with Ang II (100 nM, 24 h) or saline after (**A**) Ad-iPLA_2_β (500 MOI) and Ad-tet-on (2000 MOI), and/or doxycycline (1 µg/ml); (**B**) saline or inhibitors of lipoxygenase (NDGA, 30 µM), or cyclooxygenase (Indomethacin, 50 µM) or P450 cytochrome C (ODA, 10 µM). (**C**) Lox−/− or Wt VSMCs stimulated with Ang II or saline. n = 3–4, *: p<0.05, **: p<0.01, ***: p<0.001 one-way ANOVA.

Next, we examined the effect of iPLA_2_β overexpression on Ang II-induced vascular smooth muscle hypertrophy in cultured VSMC. We found that exogenous overexpressed iPLA_2_β was gradually lost during the passage of VSMC isolated from the iPLA_2_β-Tg mice; therefore, we overexpressed iPLA_2_β by doxycycline inducible adenoviral vector mediated gene transfer as previously described [11], [18] and achieved an 11-fold increase in iPLA_2_β protein expression (data not shown). Ang II-induced [^3^H]-leucine incorporation was significantly enhanced by iPLA_2_β overexpression to 1.73-fold (Fig. 5A). As a control, neither doxycycline nor adenovirus treatment alone had an effect on the basal [^3^H]-leucine incorporation (Fig. 5A). Together, these results suggest that iPLA_2_β overexpression facilitates VSMC hypertrophy.

To dissect the mechanism downstream of iPLA_2_β that exacerbates Ang II-induced VSMC hypertrophy, we examined the role of the arachidonic acid metabolites from lipoxygenase, cyclooxygenase or P450 pathways on [^3^H]-leucine incorporation. The inhibition of lipoxygenase by NDGA nearly abolished both basal and Ang II-induced [^3^H]-leucine incorporation (Fig. 5B), whereas, inhibition of cyclooxygenase by Indomethacin had no effect. P450 inhibition by ODA significantly attenuated Ang II-stimulated [^3^H]-leucine incorporation (Fig. 5B). VSMC isolated from 12/15 lipoxygenase knockout (lox−/−) mice also showed a significant attenuation of Ang II stimulated [^3^H]-leucine incorporation (Fig. 5C), verifying the essential role of iPLA_2_β and the 12/15 lipoxygenase pathway in Ang II-induced VSMC hypertrophy.

### iPLA_2_β overexpression promotes Ang II-induced c-Jun phosphorylation via the 12/15 lipoxygenase metabolites

**Figure 6:**
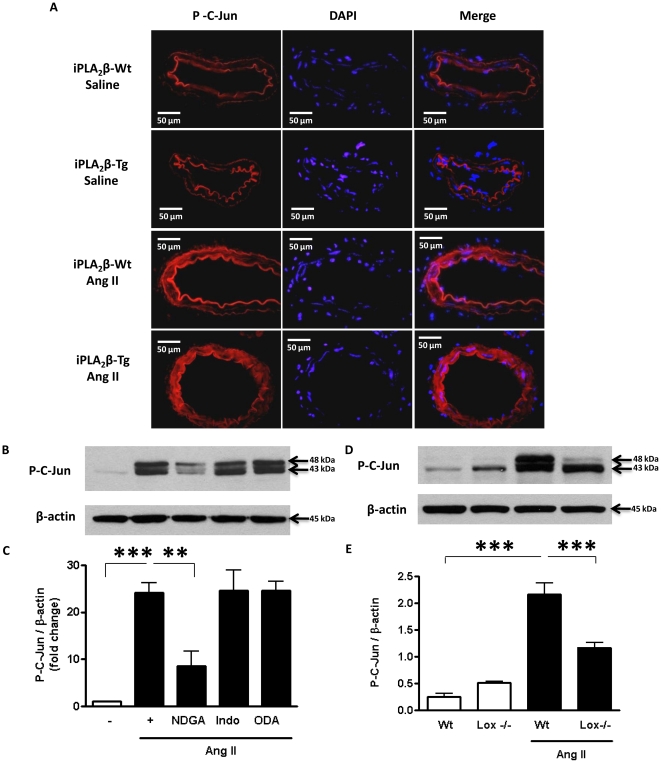
iPLA_2_β overexpression promotes Ang II-induced c-Jun phosphorylation via the 12/15 lipoxygenase pathway. (**A**) iPLA_2_β-Wt and iPLA_2_β-Tg mice were infused with Ang II (1000 ng/kg/min, 14 days) or saline and 2^nd^ order branch of mesenteric arteries VSMC were isolated and embedded in OCT. Vessel sections were stained with anti-p-c-Jun antibody and DAPI. (**B**) and (**D**) are representative western blots, (**C**) and (**E**) are quantifications of the blots, of c-Jun phosphorylation in aortic VSMCs after the same treatments as in Fig. 3 B and C. n = 3–4, *:p<0.05, **: p<0.01, ***: p<0.001 one-way ANOVA.

c-Jun phosphorylation has been demonstrated to be required for Ang II-induced VSMC hypertrophy. We therefore further investigated whether iPLA_2_β overexpression exacerbates Ang II-induced c-Jun phosphorylation by immunohistochemistry using mesentery artery sections from iPLA_2_β-Wt and iPLA_2_β-Tg mice. Ang II infusion enhanced the staining in both the iPLA_2_β-Wt and iPLA_2_β-Tg mouse sections (Fig. 6A). Moreover, the staining in the iPLA_2_β-Tg mice was much higher than that in the iPLA_2_β-Wt mice (Fig. 6A), indicating that iPLA_2_β overexpression promotes Ang II infusion-induced c-Jun phosphorylation.

**Figure 7:**
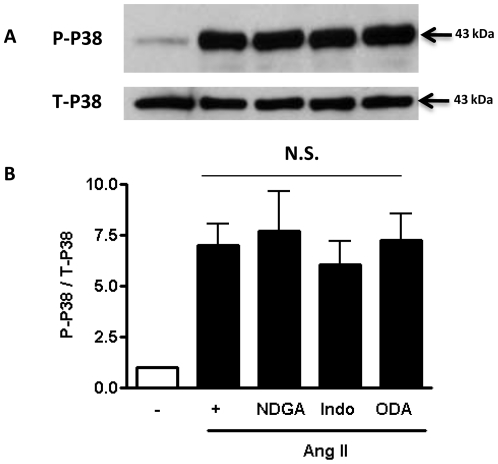
Lack of effects of inhibiting arachidonic acid metabolism on Ang II induced p38 MAPK activation. VSMC were isolated from the aortas of iPLA_2_β-Wt mice and incubated with inhibitors of lipoxygenase (NDGA, 30 µM), cyclooxygenase (Indomethacin, 50 µM) or P450 cytochrome C (ODA, 10 µM) and stimulated with Ang II (100 nM) or saline for 1 h. Then the total- or phosphorylated p38 MAPK was determined by Western Blot. n = 3–4.

To further clarify the mechanism downstream of iPLA_2_β, we examined the role of the arachidonic acid metabolites in Ang II-induced c-Jun phosphorylation. The results demonstrate that inhibition of lipoxygenase by NDGA, but not inhibition of cyclooxygenase by indomethacin or P450 by ODA significantly attenuated Ang II-induced c-Jun phosphorylation (Fig. 6B, C). A significant attenuation of Ang II-stimulated c-Jun phosphorylation was also observed in VSMC isolated from 12/15 lipoxygenase knockout (lox−/−) mice (Fig. 6D, E), indicating a critical role of the 12/15 lipoxygenase pathway in Ang II-induced VSMC hypertrophy. Interestingly, the Ang II-induced p38 MAPK phosphorylation was not affected by any of the arachidonic acid metabolism enzyme inhibitors tested (Fig. 7A, B).

### Ang II increases endogenous iPLA_2_β protein expression in VSMC

**Figure 8:**
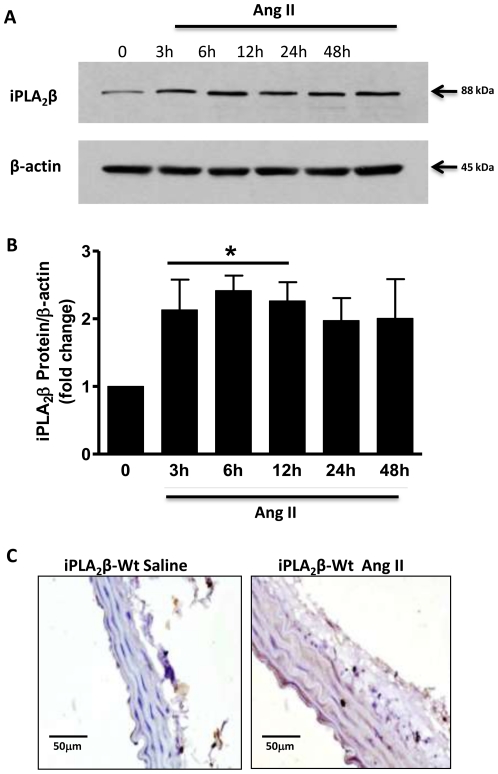
Ang II enhances iPLA_2_β protein expression in cultured VSMC and *in-vivo*. 24 h serum-deprived rat aortic VSMC were stimulated with Ang II (100 nM) for various time intervals as indicated. iPLA_2_β protein abundance was determined by Western Blot (**A** and **B**). Representative immunohistochemical images of iPLA_2_β protein staining in C57/B6 thoracic aorta from iPLA_2_β-Wt and iPLA_2_β-Tg mice infused with saline or Ang II (500 ng/kg/min, 14 days) (**C**). n = 4–5; *: p<0.05 by one-way ANOVA analysis.

To further explore a possible role of endogenous iPLA_2_β in Ang II's effects, we determined whether iPLA_2_β protein is up-regulated by Ang II *in-vitro* and *in-vivo*. In cultured iPLA_2_β-Wt VSMC, Ang II treatment significantly increased iPLA_2_β protein expression within the time frame from 3 h to 48 h (Fig. 8A–B). *In-vivo*, Ang II infusion enhanced iPLA_2_β protein expression in the thoracic aorta as indicated by immunohistochemical analysis (Fig. 8C, note brown color accumulation in the media layer).

## Discussion

In the present study, we investigate the role of up-regulated smooth muscle iPLA_2_β in Ang II-induced VSMC hypertrophy, vascular remodeling and hypertension. Several novel findings are reported here. First, using a smooth muscle specific iPLA_2_β-Tg mouse model, we find that overexpression of iPLA_2_β in smooth muscle exacerbates Ang II infusion-induced blood pressure increase and vessel remodeling. Second, we demonstrate that the up-regulated iPLA_2_β acts via the 12/15 lipoxygenase pathway to induce c-Jun phosphorylation and to promote Ang II-induced VSMC hypertrophy. Third, Ang II up-regulates iPLA_2_β protein in cultured VSMCs and *in-vivo* during Ang II-infusion.

Metabolites of free arachidonic acid produced by the three major pathways, cyclooxygenase [27], [28], lipoxygenase [29], [30] and cytochrome P450 [31] have been demonstrated to play a role in hypertension. Different classes of phospholipase A_2_ may be responsible for the release of arachidonic acid, which is rate-limiting. Among these phospholipases, the cytosolic calcium-dependent cPLA_2_ has been shown to play an important role in the development of hypertension induced by chronic NO inhibition [32]. However, to the best of our knowledge, whether the cytosolic calcium-independent phospholipase A_2_, iPLA_2_β, participates in hypertension has not been reported. The current study for the first time demonstrates that iPLA_2_β overexpression in smooth muscle does not alter basal blood pressure, but exacerbates Ang II infusion induced hypertension and vascular remodeling. The lack of effect on basal blood pressure by iPLA_2_β overexpression is likely due to the fact that under resting conditions iPLA_2_β is presumably bound to calmodulin thus catalytically inactive [13]. Upon Ang II stimulation, iPLA_2_β is dissociated from calmodulin and activated therefore exacerbating Ang II infusion-induced blood pressure increase. Several lines of evidence suggest that the enhanced Ang II infusion-induced blood pressure increase is specifically caused by iPLA_2_β overexpression in vascular smooth muscle. First, neither the locomotor activity nor heart rate is increased in the iPLA_2_β-Tg mice (Fig. 1D, F), suggesting that locomotor activity and heart rate are not responsible for the exaggerated increase in blood pressure in response to Ang II-infusion. Second, among the diastolic, systolic and mean arterial pressure, the diastolic pressure exhibited the most enhancement by iPLA_2_β overexpression (Fig. 1). It is established that enhanced diastolic pressure is associated with an increase in peripheral resistance, whereas, enhanced systolic pressure is reliant on an increase in large conduit vessel stiffness and stroke volume [33]. The selective diastolic pressure enhancement observed in iPLA_2_β-Tg mice, in response to Ang II infusion, suggests that it is the increase in resistance of the vasculature that accounts for the enhanced blood pressure response. Third, morphometric analysis of the mesentery arteries reveals that the media thickness and media∶lumen ratio are significantly increased in the iPLA_2_β-Tg. This enhanced vessel remodeling in small arteries is expected to increase peripheral resistance and thus contributes to the enhanced diastolic blood pressure observed in the iPLA_2_β-Tg mice. Finally, the systolic blood pressure was similar in the iPLA_2_β-Tg and control mice. This is consistent with the observation that there was no significant difference in the aorta elastin and collagen content, which implies that the stiffness of the conduit vessels is similar in the iPLA_2_β-Tg and iPLA_2_β-Wt mice.

Phospholipase A_2_ and arachidonic acid metabolites are involved in hypertension caused by various mechanisms; however, it is interesting to note that blood pressure under basal physiological conditions was not affected by iPLA_2_β smooth muscle overexpression (present study), or by deleting cPLA_2_ [34] or 12/15 lipoxygenase [30]. This suggests that the arachidonic acid metabolites do not alter basal blood pressure homeostasis, but play a significant role in pathological hypertension induced by Ang II infusion (present study), renal artery occlusion, or blockade of NO [30], [35]. These observations raise the possibility that the phospholipase A_2_/arachidonic acid pathway is a therapeutic target for selective lowering of blood pressure under pathological conditions without disturbing normal physiological blood pressure.

The exacerbation of vascular remodeling in iPLA_2_β-Tg mice can be a consequence in response to increased blood pressure or an enhancement of Ang II's direct effect on vasculature. Our results suggest that increasing iPLA_2_β enhances Ang II signaling in the vasculature. In cultured VSMC, we found that iPLA_2_β overexpression enhanced Ang II-induced [^3^H]-leucine incorporation, indicating promotion of Ang II's direct effects on VSMC account for, at least in part, the exacerbation of vascular remodeling. Increased VSMC proliferation and/or hypertrophy may underlie the enhanced vascular remodeling. Our results demonstrate selectively enhanced hypertrophy in the absence of detectable alterations of proliferation in both mesenteric arteries and descending aortas in iPLA_2_β-Tg mice. This further suggests that iPLA_2_β overexpression amplifies Ang II mediated hypertrophic pathways and is consistent with the observations of others who found that Ang II selectively induces hypertrophy in cultured VSMCs [36] and *in-vivo* in the descending aorta [37].

Among the elaborate signaling network activated by Ang II, the lipoxygenase [38], c-Jun NH2-terminal kinase (JNK) [39] and p38 MAPK [40] pathways are known to be essential mediators of Ang II induced VSMC hypertrophy. This study demonstrates that iPLA_2_β overexpression potentiates Ang II-induced VSMC hypertrophy via activation of the lipoxygenase pathway since [^3^H]-leucine incorporation was inhibited by NDGA (Fig. 5B) or by genetic deletion of 12/15 lipoxygenase (Fig. 5C). Interestingly, only the JNK activation, as indicated by increased c-Jun phosphorylation (Fig. 6B–E), but not p38 MAPK (Fig. 7A,B) is affected by inhibition of the 12/15 lipoxygenase pathway. This is consistent with the report that p38 MAPK is upstream of and required for iPLA_2_β activation induced by thrombin [16].

In addition to iPLA_2_, cPLA_2_ has been shown to be activated by Ang II [41], [42] and is required for Ang II-induced arachidonic acid release [43] and hypertrophy [44] in VSMC. The relationship between the cPLA_2_ and iPLA_2_ in the regulation of arachidonic acid release and cell hypertrophy remain to be established. It is conceivable that iPLA_2_ and cPLA_2_ act in a sequential manner. iPLA_2_ has been demonstrated to play a critical role in store-operated calcium influx [45] and that is crucial for cPLA_2_α activation [35]. Indeed, iPLA_2_ has been shown to mediate the early phase whereas cPLA_2_ mediates the late phase of arachidonic acid release in response to thapsigargin and ionophore A23187 in VSMCs [17].

Emerging evidence suggests that iPLA_2_β protein level is increased under disease conditions including ischemia [6], Type I diabetes mellitus in pancreatic β-cells [23] and vascular tissues [11], in addition to being regulated by complex post-translational modifications. Whereas, thrombin activation of iPLA_2_ is not associated with iPLA_2_ protein level alterations [16], the current study demonstrates that Ang II activation of iPLA_2_β is associated with up-regulation of iPLA_2_β protein in cultured VSMC and *in-vivo* in the vascular tissue (Fig. 8A–C). This is the first evidence that a G-protein-coupled-receptor agonist can up-regulate iPLA_2_β protein, suggesting that the regulation of iPLA_2_β protein is agonist specific. However, whether iPLA_2_β protein up-regulation is required for Ang II infusion induced hypertension requires further investigation using iPLA_2_β knockout mice.

In summary, the present study is the first to demonstrate a novel role of up-regulated vascular smooth muscle iPLA_2_β in Ang II-induced hypertension, vascular remodeling and VSMC hypertrophy. This effect of iPLA_2_β is mediated via the lipoxygenase pathway, which induces JNK activation. Ang II stimulation up-regulates iPLA_2_β protein expression and these results suggest that up-regulated iPLA_2_β has significant effects on the vasculature.
